# Psychophysical Estimates of Frequency Discrimination: More than Just Limitations of Auditory Processing

**DOI:** 10.3390/brainsci3031023

**Published:** 2013-07-05

**Authors:** Johanna G. Barry, Benjamin Weiss, Beate Sabisch

**Affiliations:** 1 MRC Institute of Hearing Research, University Park, Nottingham, NG7 2RD, UK; 2 Max Planck Institute for Human Cognitive and Brain Sciences, Leipzig, 04103, Germany; E-Mails: benjaminweiss.psy@googlemail.com (B.W.); sabisch@majobo.de (B.S.)

**Keywords:** language and literacy disorders, auditory processing, frequency discrimination, task design, individual differences, musical training, auditory sensory memory, verbal short-term memory, nonword repetition

## Abstract

Efficient auditory processing is hypothesized to support language and literacy development. However, behavioral tasks used to assess this hypothesis need to be robust to non-auditory specific individual differences. This study compared frequency discrimination abilities in a heterogeneous sample of adults using two different psychoacoustic task designs, referred to here as: 2I_6A_X and 3I_2AFC designs. The role of individual differences in nonverbal IQ (NVIQ), socioeconomic status (SES) and musical experience in predicting frequency discrimination thresholds on each task were assessed using multiple regression analyses. The 2I_6A_X task was more cognitively demanding and hence more susceptible to differences specifically in SES and musical training. Performance on this task did not, however, relate to nonword repetition ability (a measure of language learning capacity). The 3I_2AFC task, by contrast, was only susceptible to musical training. Moreover, thresholds measured using it predicted some variance in nonword repetition performance. This design thus seems suitable for use in studies addressing questions regarding the role of auditory processing in supporting language and literacy development.

## 1. Introduction

Many children and adults with impairments in language or literacy often also perform poorly on tasks assessing auditory processing abilities [1,2,3,4]. Such observations have led to the hypothesis that efficient auditory processing supports normal language and literacy development [2,5,6]. However, there is considerable debate about what aspect of auditory processing is relevant. More broadly, there is also debate about whether in fact auditory processing deficits are causal to delayed language or literacy development. The relationship between these different abilities may simply reflect the presence of a third underlying factor which is important for both [7]. It is difficult to exclude this possibility since few tasks are pure measures of the skills they are designed to assess. In the case of behavioural measures of auditory processing where the minimum difference between two stimuli is estimated (*i.e.*, JND: just noticeable difference), task performance not only reflects auditory abilities, but also factors associated with the design of the task and its susceptibility to a broad range of other differences specific to the individuals doing it. Understanding how these individual differences interact with task design to predict thresholds measured is important for interpreting findings from studies addressing questions of clinical or developmental interest.

Interest in understanding more about the relationship between auditory processing and language and literacy development was stimulated by some early seminal findings published by Tallal and colleagues [1,2]. They showed how children with impairments in language and literacy had particular difficulties in indicating the order of occurrence of two tones differing frequency (high *versus* low) when separated by less than 300 ms. They concluded that the children had a deficit in auditory temporal processing which was causal to their learning delays. These findings were highly influential, but they were not reliably replicated in subsequent studies [8], resulting in considerable debate about what else could have been tested in addition to temporal processing.

The studies into temporal processing relied on the assumption that all participants were equally good at discriminating between pure tones. However, if the task is modified to exclude individual differences in frequency discrimination [3], only a very small minority of reading disabled listeners continue to demonstrate difficulties with processing rapidly presented tones. As a consequence of findings such as these and others, there is now also considerable interest in evaluating the role of frequency discrimination deficits in language and literacy impairments.

Frequency discrimination deficits are relatively reliably associated with language and literacy impairments [4,9,10,11,12,13], but it is still not clear that they are causal to them. Arguments for a causal relationship are weakened by two observations. First, though deficits in frequency discrimination are frequently observed for groups of participants with language or literacy difficulties, not all individuals in the group will have these deficits. Instead, conclusions reflect the influence of a relatively large minority of poor performers [14]. Secondly, even among groups of participants with no language learning difficulties, individuals will be observed with frequency discrimination thresholds outside the normal range. These two observations together cast doubt on the hypothesis that language or literacy impairments are caused by a deficit in frequency discrimination alone (though it may represent a risk factor for them [15]). 

Apart from the matter of causality, these observations also highlight the problem of individual differences in task performance. Developmental or clinical populations typically demonstrate broad ranges of individual differences in auditory processing abilities [16] which are particularly marked for frequency discrimination [17,18]. This suggests, for reasons that are still not understood, that in addition to auditory abilities the process of comparing two (or more) tones stresses other abilities located at the level of the individual.

When assessing auditory processing behaviourally, three main factors interface to affect the final threshold (JND) measured. These are: (1) the ability to efficiently process the stimulus of interest (*i.e.*, auditory processing ability), (2) the design of the psychoacoustic task (*i.e.*, stimulus type, presentation format, and task requirements), and (3) the cognitive characteristics specific to the individual doing the task. In typical psychoacoustic studies, which aim to determine the limits of the auditory system, effects due to the individual, or to the task design are minimised by providing intensive pre-test training. Such a high level of training is neither feasible, not desirable in studies designed to address questions of clinical or developmental interest. Instead, it is implicitly assumed (often after balancing for age, IQ, and gender) that the psychoacoustic task is sufficiently robust to non-auditory differences, to permit a reliable comparison of auditory abilities across groups. However as Hirsh and Watson [19] note this assumption is not necessarily valid and any auditory-specific effects can be significantly masked by a combination of non-auditory factors interacting to a greater or lesser extent with each other and with the task used. Thus, stimuli vary in their susceptibility to pre-existing auditory experiences among individuals, e.g., musicians have better frequency discrimination abilities than the normal population [20]. Tasks vary in the intellectual, linguistic, or cognitive demands made on participant [21,22]. Individuals vary in their susceptibility to training effects inherent in task performance, as well as their auditory experiences, IQ and motivation [18], the strategies that they use to support discrimination judgments, their capacity to maintain attention [23], and their ability to efficiently allocate attentional resources to incoming auditory stimuli [24]. All these factors are exacerbated in developmental and clinical studies where participants are typically heterogeneous on a range of dimensions including, education, IQ, and musical training. Thus other processes, not necessarily stimulus-related, but specific to the individual may underlie some of the variance in auditory processing observed across tasks, possibly to the extent of exaggerating observed group differences relevant to the clinical or developmental question that motivated the study [25]. It is thus important to understand how stimulus, task, and individual interact to result in an observed discrimination threshold. More broadly, given the heterogeneity of individuals with language and literacy impairments, task designs need to be identified which are minimally susceptible to non-auditory differences among individuals. There is thus real value in comparing heterogeneous groups with a multiplicity of educational, social and auditory experiences on different tasks assessing the same aspect of auditory processing.

### Task Design to Assess Frequency Discrimination

The two-interval (2I) design (*i.e.*, comparison of a target stimulus with one standard stimulus) is one of the most commonly used designs for assessing auditory abilities in language and literacy impairments. When testing frequency discrimination, individuals are either asked to indicate for example, the order of occurrence of the higher relative to the lower tone, or they are asked to indicate if the two tones are the “same” or “different”. Some form of adaptive procedure is then used to progressively decrease the difference between standard and target tones until threshold is reached.

The relative merits of a procedure can be defined in terms of threshold estimation efficiency (*i.e.*, for a given number of trials and step size, the precision of estimation and the tightness of distribution of observations for a given sample size), as well as the resistance to bias of a particular procedure. Assessed in these terms, 2I designs have been shown to be more prone to bias (*i.e.*, they underestimate thresholds) as well as being less efficient at threshold estimation than designs based on three (3I) or four (4I) intervals (*i.e.*, one target tone compared with either two (3I) or three (4I) standard tones) [26]. Moreover, the amount of bias in threshold estimate observed using 2I designs is more dependent on choice of step size than it is for 3I or 4I designs. 

Apart from the technical issues associated with threshold estimation, 2I designs have also been criticised for placing significant demands both on early stimulus encoding [12], and higher cognitive and linguistic abilities [11,22,24] as well as being open to biased responding (*i.e.*, preferentially responding either “same” or “different”) [24]. In this study, we compared frequency discrimination abilities across three heterogeneous groups of adults using two tasks which were developed to address different aspects of the difficulties with the standard 2I design.

The first task was developed by France, Rosner, Hansen *et al.* [12] following the observation [25] that participants with literacy difficulties performed almost as well as normal readers on frequency discrimination when the task design incorporated three comparison tones (*i.e.*, a 4I design). France and colleagues hypothesised that the poorer performance of participants with language and literacy impairments on standard 2I tasks reflected noisy early encoding of auditory inputs, which made subsequent comparisons across tones more difficult. To reduce the impact of this effect, a stream of six standard stimuli (6A) was incorporated prior to the presentation of the target stimulus (X) resulting in a 2I_6A_X design. This design modification resulted in a marked reduction in variance in frequency discrimination thresholds for all participants, particularly those with reading difficulties. These observations provided support for the hypothesis that this latter group had problems with early encoding of the standard stimulus, and at the same time effectively solved these problems. Moreover, once the problem of noisy early encoding was addressed, the inter-stimulus interval (ISI) between final standard (A) and target (X) could be manipulated to further demonstrate group differences in durability of auditory sensory memory which associated with digit span in the reading disabled group. Short-term memory is thought to rely on inputs from auditory sensory memory [27], and these findings potentially explained deficits in this cognitive capacity in individuals with language or literacy difficulties [28].

In contrast with the 2I_6A_X task, the second task of interest in this study was based on a 3-interval, two alternative forced choice design (3I_2AFC). The focus of this design was two-fold: (1) to minimise the potential for biased responding which is available with the standard 2I design, and (2) to minimise linguistic and cognitive demands by making the task an odd-ball task. This design, however, potentially taxes short-term memory, since the listener has to encode and compare three stimuli. To minimise these effects, in the design used here, the second interval was obligatorily reserved for the standard tone (X), so that the target tone, occurring in either the first (A) or the third (B) interval, could be directly compared with it. This design—a 3I_2AFC, AXB design—has been successfully used to assess frequency discrimination in children as young as six years of age [29]. Though, unlike the results reported for the 2I_6A_X design, considerable individual variation in performance is typically observed [9,29]. This suggests that the task is more susceptible than the 2I_6A_X task to non-auditory specific differences among individuals.

To our knowledge, the two task designs have yet to be directly compared with the same groups of participants. The aim for this study was therefore to compare frequency discrimination on the 2I_6A_X and 3I_2AFC tasks for three groups of adults varying in education, socioeconomic background and musical experience. The three groups were recruited as part of a larger study on heritability of language learning difficulties. Two groups were defined according to their relationship to a child proband as either; parent of a child with language impairment (Par-SLI) or parent of a typically developing child (Par-TD). Their performance on the two psychophysical tasks was compared with a group of students. 

The primary question was: Do the two designs used to assess frequency discrimination differ in their susceptibility to individual differences external to the task? We predicted a smaller range of individual variation on the 2I_6A_X, which would reflect a reduced susceptibility to individual differences in the factors assessed in the study. 

Secondly we asked: What if any of the factors assessed (*i.e.*, nonverbal IQ (NVIQ), socio-economic status (SES: assessed as a combination of educational and employment background), or musical experience (*i.e.*, active training and passive listening) were particularly important for predicting performance variance?

Finally, we asked: what if any relationship exists between nonword repetition and frequency discrimination as assessed using the two psychophysical tasks? Our interest in nonword repetition reflected the fact that it is thought to probe verbal short-term memory (VSTM) [30]—a key support for language development [28,31]. Efficiency of VSTM functioning is thought to depend on auditory sensory memory [32] and this final aim reflected an interest in ultimately using the 2I_6A_X task to investigate the extent to which verbal short-term memory deficits developed out of deficits in auditory sensory memory. Auditory sensory memory is specifically tested in the longest ISI (1000 ms) condition of the 2I_6A_X task [12]. We therefore predicted a relationship between nonword repetition and frequency discrimination thresholds observed in this condition.

## 2. Results

### 2.1. Assessment of Task Specific Variations in Frequency Discrimination Abilities

**Figure 1:**
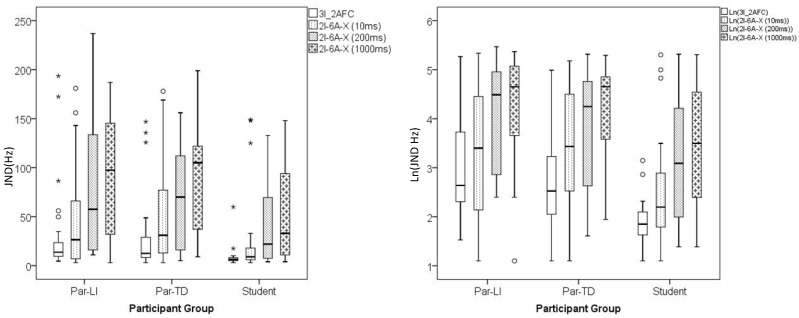
Box plot summaries of JNDs for the three groups of participants on the two psychophysical tasks. The left panel presents the raw data, while the right shows the transformed data. In the boxes, the black bars indicate median scores. The top to bottom edges of the boxes incorporate the interquartile range of performance, with the whiskers indicating the range of scores observed for each measure. Outliers are identified as falling outside 1.5 (○) and 3 (*) times the box length from the upper or lower edges of the boxes.

Figure 1 and Table 1 summarise performance of the three groups of participants on the two psychophysical tasks (2I_6A_X *versus* 3I_2AFC).

**Table 1:** Summary of raw and natural log (ln) transformed Just Noticeable Differences (JNDs) (means, standard deviations) as estimated from the 3I_2AFC and 2I_6A_X paradigms. Results from one-way ANOVAs comparing the means for the transformed data are reported. * *p* < 0.05 indicates a significant group difference.

Paradigm	ISI (ms)	Par-LI	Par-TD	Students	ANOVA
*Raw scores*					
JND_2I_6A_X_	10	47.9 (51.6)	57.1 (56.6)	24.7 (41.5)	
	200	81.0 (65.0)	73.2 (51.9)	36.9 (40.2)	
	1000	93.4 (57.3)	98.8 (60.4)	50.9 (47.0)	
JND_3I_2AFC_	500	46.9 (62.9)	26.3 (36.2)	10.4 (12.6)	
*Transformed*					
Ln(JND_2I_6A_X_)	10	3.28 (1.38)	3.49 (1.19)	2.62 (1.22)	*F*(2, 73) = 1.03
	200	4.00 (1.07)	3.84 (1.17)	3.11 (1.27)	*F*(2, 73) = 0.44
	1000	4.22 (1.05)	4.22 (1.06)	3.47 (1.25)	*F*(2, 73) = 0.97
Ln(JND_3I_2AFC_)	500	3.09 (1.16)	2.73 (0.98)	2.03 (0.69)	*F*(2, 84) = 4.02 *

First, regardless of group, there is considerable individual variation in discrimination thresholds for both task designs and for each ISI in the 2I_6A_X task. Though contrary to prediction, individual variations in JND are less marked for the 3I_2AFC compared to any ISI condition in the 2I_6A_X task.

To explore the differences among the two tasks, the data from the 2I_6A_X task for the ISI = 200 ms condition were entered into a repeated-measures ANOVA with the data from the 3I_2AFC task. This ISI condition was most similar to that of the 3I_2AFC task. Confirming the impression that participants obtained better overall JNDs with the 3I_2AFC than the 2I_6A_X task, a significant effect was observed for Task (*F*(1, 73) = 57.88, *p* < 0.001, η^2^ = 0.442). There was also a significant main effect for Group (*F*(2, 74) = 7.65, *p* < 0.001, η^2^ = 0.173, β = 0.879) reflecting both lower JNDs and reduced individual variation in performance in the student group for both tasks.

The mean thresholds for the 2I_6A_X task increase with increasing ISI in all three groups. To investigate this last effect further, the data from this task were entered into a repeated-measures ANOVA with ISI (10, 200, 1000 ms) as the within-subjects measure and Group (Par-TD, Par-LI, Student) as the between-subjects measure. Mauchly’s test of sphericity was significant (*p* = 0.007) and degrees of freedom were adjusted using Greenhouse-Geisser correction factors. A significant effect was observed for ISI reflecting the progressive increase in discrimination threshold with increasing ISI (*F*(1.78, 131.32) = 58.89, *p* < 0.001, η^2^ = 0.443, ε = 0.887). *Post hoc* Bonferroni tests indicated that mean thresholds for all three ISIs differed significantly from each other. There was also a significant main effect for Group (*F*(2, 74) = 3.486, *p* < 0.05, η^2^ = 0.086, β = 0.635), reflecting the lower thresholds observed among the student group. The two parent groups had comparable JNDs. There was no significant Group x ISI interaction (*i.e.*, JNDs for all three groups were similarly affected by increasing ISI). 

### 2.2. Training Effects on JND Estimation in the 2I_6A_X Task

Neither the large variability in JNDs measured using the 2I_6A_X task, nor the lower JNDs for the 3I_2AFC task, were predicted at the outset of the study. Initial piloting with students suggested that the 2I_6A_X task was procedurally more difficult than the 3I_2AFC task. The study protocol was consequently set so that the 3I_2AFC task was always presented first. Then the ISI = 400 ms condition was used as a training session for the 2I_6A_X task and each condition started with four condition specific training trials. This testing protocol was expected to minimise any training effects on performance for the 2I_6A_X task. However, varying degrees of experience with the 2I_6A_X task may still have contributed to the individual variation observed, since the order of presentation of the remaining ISI conditions in the task was randomised. The data were therefore entered into a repeated-measures ANOVA, ISI (3) × Group (3) × Order (6) to test for such effects. A significant effect for ISI was found (*p* < 0.001), but there was no effect (or indeed any trend) for order of presentation of ISI condition. There was also no significant interaction between Order × Group. Thus individual differences in experience with the task did not significantly contribute to the broad individual variation observed in performance on it.

### 2.3. Task-Specific Susceptibility to Individual Differences

To investigate susceptibility of the two different task designs to individual differences in musical experience (listening and training), nonverbal IQ (NVIQ) and SES, these variables were entered as predictors (forced entry) into a series of multiple linear regression analyses with ln(JND) for each task/ISI condition as outcome measure. Initial models were optimised to retain only those predictors that significantly contributed to each outcome measure.

The data were first checked for evidence of significant multicollinearity between predictors (correlations greater than 0.8), or correlation between errors (Durbin-Watson statistic, values less than 1 or greater than 3). The effect of influential cases was assessed by checking for data points where Cook’s distances were greater than 1, Mahalanobis distances were greater than 15, or leverage values were greater than twice the average leverage value (*i.e.*, for the 2I_6A_X task > 0.08; for the 3I_2AFC task > 0.05). One participant was excluded from the 2I_6A_X dataset because of a marked bias for responding “different” (*d*′ = 2.15, criterion *c* = −0.84) resulting in very low JND estimates which contrasted with the JND observed for the 3I_2AFC task (172.5 Hz *versus*, for example, 3 Hz (ISI = 1000 ms)).

The regression weights for each analysis are summarised in Table 2. Predictors making nonsignificant contributions are shown to the right of the table. The inputs into the final models are bolded together with the amount of variance (*R*^2^) explained by each model.

**Table 2:** Standardised regression coefficients for the four different outcome measures (2I_6A_X, *n* = 73; 3I_2AFC, *n* = 86). ^a^ Values summarise the regression weights of the final model. ^b^ Nonsignificant regression weights observed during exploratory analyses but subsequently removed from the final models. ** *p* < 0.01, *** *p* < 0.001, n.s. (nonsignificant), *p* > 0.05.

	SES	Music training	R^2^	NVIQ	Music listening
2I_6A_X (10 ms)	−0.29 ^a, ^**	−0.39 ^a, ^***	0.27 ^a^	0.20, *p* = 0.09 ^b^	−0.07, n.s. ^b^
2I_6A_X (200 ms)	−0.26 ^a, ^**	−0.50 ^a, ^***	0.36 ^a^	0.03, n.s. ^b^	−0.12, n.s.^ b^
2I_6A_X (1000 ms)	−0.33 ^a, ^**	−0.50 ^a, ^***	0.43 ^a^	0.05, n.s. ^b^	−0.05, n.s. ^b^
3I_2AFC	−0.20, *p* = 0.06 ^b^	−0.32 ^a, ^**	0.16 ^a^	−0.19, *p* = 0.10 ^b^	−0.05, n.s.^ b^

SES and musical training were the only factors to significantly contribute to variance in JND estimates in the 2I_6A_X task. The amount of variance explained by these predictors increased with increasing ISI to a maximum of 43% for the longest ISI (1000 ms), as compared with an initial 27% for the shortest ISI (10 ms). The regression weights for musical training across the 200 and 1000 ms ISI conditions are equivalent. Musical training predicts more individual variation in JND in these two ISI conditions, than it does for the 10 ms ISI condition. SES explains more variation in JND for the 10 and 1000 ms ISI conditions, than it does for the 200 ms condition.

By contrast with the 2I_6A_X task, only musical training explained significant variance in JNDs for the 3I_2AFC task and the amount explained by it was considerably less than that explained by SES or musical training for any condition in the 2I_6A_X task. Overall, the 3I_2AFC task is less susceptible to individual differences in the factors assessed here than the 2I_6A_X task.

#### Effect of Different Task Requirements on Observed Threshold

If the higher thresholds and more variable performance in the 2I_6A_X task reflect the fact that it is more demanding than the 3I_2AFC task, then the participants who are least able to cope with the extra demands of the task will have the highest thresholds for it. They would therefore be expected to show the greatest amount of improvement in the easier task [21] which stresses their weaker cognitive skills less. To test this prediction, correlations were performed between threshold estimates obtained on the 2I task *versus* amount of improvement observed for the 3I task. Significant positive correlations (*p* < 0.001) were observed for all ISI conditions (ISI 10: *r* = 0.715; ISI 200: *r* = 0.724; ISI 1000: *r* = 0.731), confirming this prediction and suggesting that the 2I_6A_X task was inherently more difficult to do than the 3I_2AFC.

### 2.4. Contribution of Frequency Discrimination to Nonword Repetition

To assess contributions of frequency discrimination to verbal short-term memory, discrimination thresholds in the 3I_2AFC task and the three ISI conditions of the 2I_6A_X task were entered into a multiple linear regression analysis (forced entry), with “schooling” (proxy for vocabulary knowledge), and “music training”. This latter factor was included in the model because of the relationship to frequency discrimination performance observed in this study, and also because musical training is thought to enhance efficiency of auditory processing and hence support language learning [33].

Only two predictors explained significant variance in nonword repetition: schooling, and JNDs measured using the 3I_2AFC task. The three ISI conditions of the 2I_6A_X task demonstrated high multicollinearity (*r* ≥ 0.8) with each other which contrasted with the low correlations (<0.38) of each measure with the JNDs observed for the 3I task. None of the JNDs for any ISI condition explained significant variance in nonword repetition and they were deleted from the final model, together with musical training which also explained little or no variance in nonword repetition. Table 3 summarises the final regression model together with observations from the initial exploratory analyses.

**Table 3:** Summary of the standardised regression coefficients for the two frequency discrimination tasks (2I_6A_X and 3I_2AFC), schooling and music training for predicting nonword repetition ability. ^a^ Values indicate significant regression weights for the variables remaining in the final model. ^b^ Nonsignificant regression weights observed during early exploratory analyses. *** *p* < 0.001.

	Predictor	Schooling	3I_2AFC	*R*^2^		2I_6A_X		Music training
Outcome					10 ms	200 ms	1000 ms	
Nonword repetition		0.32 ^a, ^***	−0.31 ^a, ^***	0.26 ^a^	0.04 ^b^	−0.07 ^b^	−0.08 ^b^	0.01 ^b^

## 3. Discussion

In this study, we compared frequency discrimination in the same groups of participants for two tasks designed to address different problems with the standard 2-interval psychoacoustic task designs. Our results suggest the 3I_2AFC design is less susceptible to non-auditory specific differences among participants than the 2I_6A_X design. These findings were surprising given that this latter design is often cited as minimising problems with sensory memory trace formation [12], auditory attention [24] and formation of perceptual anchors [34]. In the following, as part of addressing the issue of task susceptibility to task external effects like musical training, we consider why different results were obtained to those reported by France *et al.* [12]. We conclude by addressing the question of whether or how frequency discrimination may support language development.

### 3.1. Performance Variability on the 2I_6A_X Task

An appealing aspect of the 2I_6A_X task design was that it did not appear to be susceptible to non-auditory specific differences among individuals [12] suggesting it could be reliably employed in studies involving heterogeneous populations. We did not, however, replicate this observation. A number of possible explanations come to mind to explain our different findings. 

Firstly, in the earlier study [12] a two-step process of threshold estimation was applied. Rough estimates were obtained of discrimination threshold and then a final threshold was estimated using an initial ΔF close to the expected final threshold together with a more refined (*i.e.*, smaller step size) adaptive staircase procedure. The small initial ΔF for the second estimation would have also limited the range in which variation could be observed and all participants would have had considerable experience on the task at the point of final threshold estimation, minimising training effects. This approach to threshold estimation, better approximates standard psychophysical procedures and it is likely that had we adopted a similar strategy, we would have observed lower thresholds and reduced individual variation across the groups. However, we were primarily interested in task designs that could be reliably applied to address clinical or developmental questions, where one rarely has the luxury of applying such techniques. 

Secondly, although France *et al.* [12] provide little information about their participants, it is clear they were high functioning. The mean IQs for both the reading disabled and normal readers were more than 1 standard deviation above the population mean. The majority of the participants were therefore likely to have been students, who, as our own data demonstrate, tend to perform better than the general population on psychophysical tasks. Moreover, they tend to be more homogeneous in terms of education and socio-economic status which will also limit the impact of such individual differences on thresholds observed. 

Thirdly, and related to the preceding point, there is an interesting literature suggesting 2I tasks stress cognitive abilities [22] more than other tasks designs [21,24,25]. We did not expect the 2I_6A_X design to be similarly demanding since the inclusion of a stream of six repeated standard tones should have minimised cognitive demands, first by enhancing sensory memory for the standard relative to the target tone and second by cueing the presentation of the target tone to enhance temporal focus of attention [24]. However, in an analogous analysis to that used by Bishop *et al.* [21] to compare backward-masking in child populations using 2I or 3I tasks, we observed how the participants who performed worst on the 2I_6A_X task demonstrated the greatest improvements on the 3I task. Moreover, despite designing our protocol to maximise experience on the 2I_6A_X task prior to testing, some participants had final thresholds that were greater than the initial starting point of Δ*F* = 160 Hz (Figure 1) though they got sufficient numbers of catch trials at this level correct. Together these observations suggest the poor performance on the 2I task was less about perceptual limitations, than about task-specific cognitive demands. This raises a question regarding why this design should be so cognitively demanding. In response to this, we can only provide the anecdotal evidence of our participants. Many commented that they found it more difficult than the 3I_2AFC task. Some noted how they had to concentrate harder when doing the task, while others commented that the target tone was always different, because it was longer as well as higher than the preceding six tones. Target and standard tones can have a variable perceptual quality particularly around threshold which contributes to the difficulty of correctly identifying the target tone. The comments of our participants suggest the presentation format for the 2I_6A_X task may have complicated the auditory decision-making process by promoting confusions in auditory percept. 

Finally, although we have focused entirely on issues to do with task design, it is also possible that differences in the stimuli may have also contributed to the variations in individual differences apparent between the two tasks. Roving of the standard frequency in a frequency discrimination task, as we did in the 2I_6A_X task, makes it cognitively more demanding, resulting in greater individual differences in performance [35]. Differences in stimulus duration may also have contributed since the tones in the 2I_6A_X task were longer than those used in the 3I_2AFC task. Our design does not allow us to assess these stimulus-specific effects, though work by Banai and colleagues suggest they may be outweighed by effects specific to the task [22].

### 3.2. Contributions to Task Performance of Different Environmental Factors

Environmental effects such as socioeconomic status (SES) and more particularly, active musical training, but not passive music listening, had a significant effect on threshold estimates for both the 2I_6A_X and 3I_2AFC tasks. Musical training may support frequency discrimination by developing a more sophisticated sense of how different sounds relate to each other. The SES measure used here incorporated among other things differences in education which may impact of an individual’s ability to develop different strategies to cope with varying task demands. The 2I_6A_X task was particularly sensitive to these factors with the relative impact on task performance increasing with increasing ISI. This supports our earlier conclusion, that it is a cognitively demanding task and further suggests that it became more cognitively demanding as the time interval between standard and target increased meaning listeners became increasingly reliant on other (non-auditory) skills to support processes involved in their decision-making. The increasing contribution of musical training to performance on the longer ISI conditions in the 2I task further suggests that musical training may enhance skills associated with auditory imaging [36] which in turn would support sensory memory for the standard tone. This idea is reminiscent of rehearsal mechanisms which support information storage in the Baddeley and Hitch [37] model of verbal short-term memory.

We are not the first to note how musical training makes a significant contribution to frequency discrimination abilities. Micheyl *et al.* [20] have demonstrated excellent frequency discrimination abilities in professional musicians. However, our data suggest, very little musical training is required to enhance frequency discrimination abilities, since none of the participants in this study were professionally trained. 

Bishop [15] has previously noted how exposure to music in the home was an important environmental factor for predicting performance on a test of rapid temporal processing in a study investigating auditory and cognitive abilities in twins. We did not replicate this finding; nonetheless, both our study and that of Bishop [15] demonstrate the sensitivity of the auditory system to environmental factors like musical training or listening.

### 3.3. Relationships between Frequency Discrimination and Nonword Repetition

Auditory sensory memory is hypothesised to support verbal short term memory and we were ultimately interested in assessing the suitability of the 2I_6A_X task for further studying this component of verbal short-term memory especially since France *et al.* [12] had reported a relationship between frequency discrimination thresholds for the 2I_6A_X task and digit span—a measure of short-term and working memory. We therefore predicted that we would also observe a relationship (particularly for the long (1000 ms) ISI condition) with the nonword repetition task (another measure of short-term memory). However, no evidence of a relationship with nonword repetition was observed for any ISI condition in the 2I_6A_X task. By contrast, a small but significant association was observed between nonword repetition and JNDs estimated using the 3I_2AFC task. These observations add to a body of literature reporting similar such associations between frequency discrimination and performance on a range of tasks including, reading and phonological decoding skills [4,38], nonword same/different discrimination [22], as well as nonword repetition [39]. Such associations suggest frequency discrimination may impact on speech perception and hence on ability to develop language and literacy. However, we do not think the relationship is quite so direct. Gathercole and Baddeley [40] found no association with speech perception and repetition of short nonwords. In a similar vein, Rosen and Manganari [41] were unable to demonstrate a clear link between deficits in auditory processing and speech perception skills in a group of children with dyslexia. Finally, Halliday and Bishop [39] showed how, despite having deficits in frequency discrimination, children with mild to moderate hearing losses did not obligatorily demonstrate difficulties in reading or nonword repetition.

Overall, while we did observe a link between the frequency discrimination and nonword repetition using a task that was relatively robust to the individual differences assessed in this study, our study does not rule out the involvement of a third higher cognitive capacity which is separately relevant to both nonword repetition and frequency discrimination. Similar arguments have also been put forward by Halliday and Bishop [39] and Banai and Ahissar [22]. Halliday and Bishop proposed auditory attention as a possible candidate for this third cognitive capacity, while Banai and colleagues have argued that whatever the capacity, it may not be specifically auditory in nature, and is likely to be mediated by higher level processes involving the engagement of the pre-frontal cortex which associates both with attention and with memory.

## 4. Experimental Section

### 4.1. Subjects

Eighty-nine adults with normal hearing (pure tone thresholds ≤ 25 dB HL for frequencies 500, 1000, and 2000 Hz) participated in the study. They were all native speakers of German and were subdivided into three groups: Students, parents of typically-developing children (Par-TD), and parents of children with language impairments (Par-LI). This latter group typically have poorer nonword repetition abilities [42] than is typically observed in the population as a whole, so in addition to considerable individual variation in education, socio-economic status and musical experience (both listening and training), our participant mix also incorporated a relatively broad range of nonword repetition abilities.

The student group (*n* = 21) were aged between 20 and 28 years and were studying at the University of Leipzig. They were recruited via the adult participant databank at the Max Planck Institute for Human Cognitive and Brain Sciences. Of this group, one participant was subsequently excluded because she did not satisfy our definition of normal hearing. 

The parent groups (Par-TD (*n* = 36); Par-LI (*n* = 32)) were recruited from either Leipzig or Berlin as part of a larger study into risk factors for language impairment. Group membership was based on whether the participant’s child met definitional criteria for either typical development or language impairment. The criteria were normal hearing and normal NVIQ [43]. Additionally for language impairment the child had to have a history of language difficulties and perform below 1.5 s.d. on at least one of two subtests (Comprehension and Imitation) from the *Heidelberger Sprachentwicklungstest* battery (HSET) [44]. For typical development, the child had to have no history of language learning delays and standard scores no less than 1 s.d. below the mean on the two HSET subtests.

### 4.2. Behavioural Tests

#### 4.2.1. Nonverbal IQ

NVIQ was assessed using the *Hamburg-Wechsler-Intelligenztest für Erwachsene* (HAWIE: [45]). This test comprises five subtests: (1) block design, specific patterns are created using blocks; (2) picture-ordering, a series of pictures are assembled to make a coherent story; (3) picture completion, the missing element in a picture must be identified; (4) figure creation, pieces of a puzzle are assembled to create specific figures; (5) number-symbol association test, numbers are translated into specific symbols as quickly as possible. Scores from the five-subtests are summed and converted to a standard score (mean = 100 ± 15). 

#### 4.2.2. Nonword Repetition

The nonword repetition task [46] consisted of 19 nonwords ranging in length from 2 to 5 syllables. The nonwords conformed to the phonological requirements of German. Up to 2 syllables per word included a consonant cluster. The words were recorded by a native German-speaking woman who was requested to locate word stress according to what felt natural for her. Typically, stress was located on the penultimate syllable for the 2, 3, and 4-syllable nonwords, and on the third syllable for 5-syllable nonwords.

The nonwords were presented over headphones (Sennheiser HD 202) at a comfortable listening level (70 dB SPL). Participants were required to repeat them as accurately as possible, and were awarded one point for each correct syllable (maximum score 64). 

All scoring was done on-line then checked off-line by a second scorer. There are some dialectal differences in pronunciation between *Hochdeutsch* (the dialect used for recording) and the dialects of some of the speakers. Criteria were set prior to scoring to ensure consistency of scoring decisions, and to ensure participants were not penalized for regional variations in pronunciation.

### 4.3. Measures of Socioeconomic Status and Auditory Experience

Measures of socioeconomic status (SES) and auditory experience were developed using responses from two questionnaires. One questionnaire obtained information about the participants’ auditory (specifically musical) background, while the second obtained information about their schooling and employment experience. Measures were then developed as described below to locate participants into different bands according to musical and socioeconomic background. 

#### 4.3.1. Musical Background

Music experience was subdivided into: (1) Musical training received, which was broken down into instrumental, dance, and vocal experience with each being scored based on amount of training received (*i.e.*, none = 0, less than 2 years = 1, more than 2 years = 2); or (2) Music listening experience, which was based on amount of musical listening during the week (*i.e.*, none = 0, 2 h or less = 1, 3 h or more = 2), and frequency of attendance at classical music concerts (*i.e.*, never = 0, sometimes = 1, often = 2). Scores for each response were summed and grouped into three bands defined as, “little”, “some” or “lots of” experience for each of training and listening experience.

#### 4.3.2. Socioeconomic Status

A measure of socioeconomic status (SES) was developed based on a combination of three variables: level of schooling (score 1–4), professional training (score 1–7), and current employment (score 1–3). Scores were summed for each participant and they were grouped into three bands: “low”, “middle” and “high”. 

Vocabulary knowledge is known to impact on nonword repetition performance [47,48,49]. We could not directly measure differences in vocabulary knowledge, but were able to use schooling as a proxy for it, since there are three different school systems in Germany which differ in the kind of education they provide. The *Hauptschule* focuses on technical education on only provides a basic secondary school education. Better marks are required for entry into the so-called *Realschule*, and *Gymnasium* school systems, where children receive a more extended education. The *Gymnasium* school system focuses on preparing students for possible entry into university.

#### 4.3.3. Summary

Table 4 summarises mean age, nonverbal IQ (NVIQ), nonword repetition scores, band of SES and band of musical experience (training and listening) for each of the three groups of participants. The student group had a higher mean NVIQ than the two parent groups who were relatively well matched on this measure. Much as previously reported within the English-speaking context [42], the Par-LI group performed significantly worse on the nonword repetition task than either the Par-TD or the student groups. Likewise, the mean SES rank for the students was significantly higher than for either group of parent. The mean SES rank for the Par-TD group was higher than that of the Par-LI, though the difference did not reach statistical significance (*p* > 0.05). With respect to music experience, the students tended to listen to more music than either group of parent, but the differences were not statistically significant.

**Table 4:** Summary of means, and standard deviations (in parentheses) for demographic and cognitive data and different environmental factors. *F*-tests from a series of one-way ANOVAs testing for differences among the three group are reported, ** *p* < 0.01, *** *p* < 0.001, n.s. = nonsignificant. Par-LI = parent of child with language impairment; Par-TD = parent of typically-developing child.

M:F	Par-LI 10:22 (*n* = 32)	Par-TD 13:23 (*n* = 36)	Student10:10 (*n* = 20)	*F*(2, 85)
Age(years)	36.74 (6.43)	36.9 (4.87)	23.80 (2.91)	
	26.3–50.5	27.9–46.6	19.1–30.0	
NVIQ	107.34 (12.3)	109.39 (12.3)	119.4 (14.1)	5.9 **
(standard score)	84–139	84–148	90–145	
NW-Rep(raw)	52.0 (5.9)	57.2 (4.0)	57.6 (2.5)	13.9 ***
	34–62	46–62	52–61	
Music training	0.28 (0.63)	0.56 (0.77)	0.58 (0.61)	n.s.
Music listening	1.13 (0.66)	1.14 (0.79)	1.53 (0.73)	n.s.
SES	1.84 (0.72)	2.14 (0.76)	2.89 (0.45)	13.9 ***

### 4.4. Frequency Discrimination Using the 2I_6A_X Task Design

The stimuli A and X were 300 ms in duration with cosine-gated 20 ms rise-fall times. The frequency of the standard (A) was roved between 480 and 519 Hz. The inter-stimulus interval (ISI) between the six A stimuli was 50 ms. There were four possible ISI duration conditions between the final A and target X: 10, 200, 400, or 1000 ms. The 400 ISI condition was treated as a training condition with the order of the remaining three conditions being randomised across participants. Four short training trials were provided prior to each new ISI condition. In two of the trials, A and X were maximally different while in the other two they were equivalent. 

The accelerated stochastic adaptive procedure [50] was used to estimate thresholds. This method has been characterised for use in practical applications involving small-samples and staircases of limited length [51]. In this paradigm, estimation of the magnitude and direction of step-change depends on the response from the previous trial, thus current step-size (*c*_current_) is changed from an initial step-size “*c*” of 48 Hz according to the formula:

*c*_current _= *c* × (*z*_prev_ − *p*_target_)/(*n*_reversals_ + 1) (1)

*c*_current_* =* step size of current trial, *c* = initial step size, *p*_target_ = target probability of correct response, *n*_reversals_* =* number of step direction changes, *z*_prev_= previous trial response.

Δ*F*_current_ = Δ*F*_prev_ − *c*_current_ (2)

Δ*F*_current_ = Standard (A) − Target (X) (difference of current trial), Δ*F*_prev_* =* Standard (A) − Target (X) (difference of previous trial). 

The initial frequency of the target (X) was set at (A + 160) Hz. This was considerably higher than the value of the target for the study described by France *et al.* [12] (*i.e.*, A + 60 Hz) but similar to that used in the 3I_2AFC task. Previous experience suggested this latter value of Δ*F* would be within the likely range of discrimination ability of the poorer performers. 

The threshold targeted was set to the 75% correct point on the psychometric function with final threshold (JND: Just Noticeable Difference) being inferred from the final trial after 10 reversals [51]. 

Catch trials were presented with a probability of 0.25 at random intervals during testing. They represented the two end points in the discrimination task, *i.e.*, Δ*F*_(X__−__A)_ = 160 Hz and Δ*F*_(X −__A)_ = 0 Hz. Feedback was provided for these trials to give participants some information regarding performance, and to enable the tester to check on-line for high false alarm and miss rates. 

Participants indicated if the target was different from the preceding six standards by pressing the left “alt” button, if they thought it was the same they pressed the right “alt” button. Visual reminders were provided to support the decision-making process.

Testing typically lasted 20 min per participant. Following France *et al.* [12], data from 5 participants, who averaged 25% or more incorrect catch trials across the three conditions (2 Par-TD and 3 Par-LI) were excluded from the final analyses.

### 4.5. Frequency Discrimination Using the 3I_2AFC Task

The pure tones for the 3I_2AFC task were 100 ms in duration with cosine-gated 10 ms rise-fall times with an inter-stimulus interval (ISI) of 500 ms between each of the three tones. The standard X was fixed at 300 Hz and the target in either the first (A) or the third (B) interval was always higher in frequency. The initial frequency for the target was 500 Hz (*i.e.*, Δ*F* = 200 Hz). Each tone was represented by a dinosaur which jumped on a box as it sounded. After choosing an interval, listeners were provided with feedback regarding the correctness of their decision. The value of ΔF was then changed using an adaptive PEST procedure (More virulent PEST, [52]). The initial step-size for decreasing the frequency of the target tone was 24 Hz to rapidly approach threshold. To more carefully measure discrimination around threshold, the step-size was increased (reversal) or decreased according to the correctness of the listener’s decision. A maximum of 8 reversals was permitted and the discrimination threshold was set to 75% correct on the psychometric function. Final threshold (JND) was defined as the mean frequency difference between target and standard tones for the last 4 reversals.

Participants typically performed the task twice with the first run being treated as a training session.

### 4.6. General Testing Protocol

A full test session lasted up to 2 h. The same general procedure was followed for all testing. There was a hearing screening, then testing began with the 3I_2AFC frequency discrimination task, followed by the nonword repetition test and ending with the 2I_6A_X test. Participants had a small rest before assessment of NVIQ.

### 4.7. Statistical Analyses

Preliminary analyses indicated normal distributions for age and NVIQ for all three groups (Shapiro-Wilk, *p* > 0.05) and a normal distribution for nonword repetition for the student and Par-LI group but not the Par-TD group. Attempts to normalise the data were unsuccessful, and the raw data were used for all subsequent analyses.

The data from two psychophysical tasks (3I_2AFC; and 2I_6A_X) were also significantly non-normally distributed with a right-skew. The data were natural log (ln) transformed to approach a more normal distribution.

## 5. Conclusions

In this study, we compared differences in frequency discrimination thresholds measured using two different psychoacoustic tasks (2I_6A_X and 3I_2AFC). We were specifically interested in whether they differed in their susceptibility to individual differences in NVIQ, socioeconomic status and musical experience, and if so which of these factors was particularly important for affecting observations. Musical experience was relevant for performance on both tasks, but the 2I_6A_X task was also susceptible to differences in socioeconomic status. This reflects the fact that, contrary to prediction, this task stressed cognitive abilities as well as auditory abilities. Overall, when studying the role of auditory abilities in intrinsically heterogeneous populations, tasks like the 3I_2AFC task are more suitable for use because they are less cognitively demanding and are hence less susceptible to effects located at the level of the individual.
